# Differentiation-Dependent Association of Phosphorylated Extracellular Signal-Regulated Kinase With the Chromatin of Osteoblast-Related Genes

**DOI:** 10.1359/jbmr.090705

**Published:** 2009-07-06

**Authors:** Yan Li, Chunxi Ge, Renny T Franceschi

**Affiliations:** 1 Department of Periodontics and Oral Medicine, University of Michigan School of Dentistry 1011 N. University Ave., Ann Arbor, MI, USA; 2 Department of Biological Chemistry, University of Michigan School of Medicine 1011 N. University Ave., Ann Arbor, MI, USA

**Keywords:** osteoblast, transcriptional factors, chromatin, osteocalcin, mitogen-activated protein kinase

## Abstract

The ERK/MAP kinase pathway is an important regulator of gene expression and differentiation in postmitotic cells. To understand how this pathway controls gene expression in bone, we examined the subnuclear localization of P-ERK in differentiating osteoblasts. Induction of differentiation was accompanied by increased ERK phosphorylation and expression of osteoblast-related genes, including osteocalcin (*Bglap2*) and bone sialoprotein (*Ibsp*). Confocal immunofluorescence microscopy revealed that P-ERK colocalized with the RUNX2 transcription factor in the nuclei of differentiating cells. Interestingly, a portion of this nuclear P-ERK was directly bound to the proximal promoter regions of *Bglap2* and *Ibsp*. Furthermore, the level of P-ERK binding to chromatin increased with differentiation, whereas RUNX2 binding remained relatively constant. The P-ERK-chromatin interaction was seen only in RUNX2-positive cells, required intact RUNX2-selective enhancer sequences, and was blocked with MAPK inhibition. These studies show for the first time that RUNX2 specifically targets P-ERK to the chromatin of osteoblast-related genes, where it may phosphorylate multiple substrates, including RUNX2, resulting in altered chromatin structure and gene expression. © 2010 American Society for Bone and Mineral Research

## Introduction

The extracellular signal-regulated kinase (ERK)–MAPK and related signal-transduction pathways have important functions in the differentiation of postmitotic cells beginning in the earliest stages of development. For example, FGF4 activation of ERK1/2 is required for embryonic stem (ES) cells to undergo neural or mesodermal differentiation; genetic deletion of *Erk2* completely blocks the FGF4 response, locking cells in a proliferative state where pluripotency markers continue to be expressed.(1) Consistent with this finding, *Erk2-*null mice exhibit severe defects in primary mesenchyme formation without major changes in cell proliferation or apoptosis.(2) MAP kinase–related pathways also control gene expression and cellular fate of more differentiated cells such as mesenchymal stem cells (MSCs) or osteoprogenitor cells in response to changes in hormone/growth factor signaling, extracellular matrix (ECM)–integrin binding, or mechanical environment.(3–10) Furthermore, the activity of individual lineage-specific transcription factors such as MYOD (muscle), SOX9 (cartilage), PPARγ (adipose tissue), and RUNX2 (osteoblasts and hypertrophic chondrocytes) are all regulated by MAP kinases.(11–14) Taken together, these studies indicate that MAP kinases can have global effects on the differentiation fate of cells during organogenesis.

Using a transgenic approach, we recently established an important role for ERK-MAPK signaling in skeletal development.(15) Mice overexpressing a constitutively active form of the MAP kinase intermediate MEK1 in osteoblasts (*Mek-sp* mice) exhibited accelerated skeletal development, whereas a dominant-negative MEK1 (*Mek-dn* mice) inhibited this process. Similarly, osteoblast-specific gene expression and mineralization were accelerated in calvarial or marrow cells cultured from *Mek-sp* mice, whereas these events were delayed in *Mek-dn* cells. Effects of MAP kinase on differentiation appeared to be mediated by the RUNX2 transcription factor in that constitutively active MEK1 was able to partially rescue the cleidocranial dysplasia phenotype associated with *Runx2* haploinsufficiency. These studies provided in vivo validation for previous cell culture work showing that the ERK-MAP kinase pathway is required for extracellular matrix (ECM)–induced osteoblast differentiation and that this pathway can stimulate RUNX2 phosphorylation and transcriptional activity.(4,13,16,17)

Although the ERK/MAPK pathway has been studied extensively in bone, the subcellular and subnuclear distribution of activated ERK during osteoblast differentiation has not been examined previously. The classic view of MAP kinase action supposes that phosphorylation events are distinct from changes in gene expression. However, more recent studies indicate that nuclear kinases may interact directly with the chromatin of target genes to control differentiation fate in response to environmental signals.(18,19) In this study, we explore this possibility in bone by examining the intranuclear localization of the terminal MAP kinase ERK1 in differentiating osteoblasts. As will be shown, the MAP kinase pathway is activated in response to ECM signals, resulting in accumulation of P-ERK in the nucleus, where, together with RUNX2, it specifically associates with osteocalcin (*Bglap2*) and bone sialoprotein (*Ibsp*) genes to induce osteoblast-specific gene expression.

## Materials and Methods

### Cell culture

Subclone 4 MC3T3-E1 (MC3T3-E1c4) cells were plated at a density of 50,000 cells/cm^2^ and maintained in growth medium containing α-Minimal Essential Medium (α-MEM; Life Technologies, Inc., Grand Island, NY, USA) supplemented with 10% fetal bovine serum (FBS; Hyclone Laboratories, Logan, UT, USA) and 1% penicillin/streptomycin, as described previously.(20) To induce differentiation, cells were cultured in the same medium containing 50 µg/ml ascorbic acid [differentiation medium (DM)]. For mineralization studies, DM was supplemented with 5 mM β-glycerol phosphate, and mineral was stained by the von Kossa method.(21) Mineralized nodules were photographed using a Nikon TS100 inverted microscope.

### Quantitation of mRNA

Total RNA was isolated from cells using TRIzol reagent (Invitrogen) and cleaned with an RNeasy Mini Kit (Qiagen). Two micrograms of total RNA was reverse transcribed using Taqman reverse transcriptase (Applied Biosystems) for first-strand synthesis. Quantitive real-time polymerase chain reaction (PCR) measurement of Ocn, Bsp, and GAPDH mRNA sequences was carried out, as described previously,(22) using predeveloped Taqman probes and an ABI PRISM 7700 sequence detector (Applied Biosystems). The real-time PCR product, mRNA expression, was calculated based on a relative standard curve and normalized to glyceraldehyde-3-phosphate dehydrogenase (GAPDH).

### Western blot measurement of Runx2 and MAP kinase

Cells were harvested in lysis buffer (10 Mm Tris, 5 mM EDTA, 0.25% Triton X-100, 150 mM NaCI, PMSF, 1 × proteinase inhibitor cocktail, Sigma). The crude membrane/nuclear fraction was isolated by centrifugation (10 minutes, 15,000 × *g*) and fractioned by SDS-PAGE using 6% to 20% TG gels. After transfer to nitrocellulose membranes, samples were incubated with RUNX2 antibody (1:500 dilution; Applied Biosystems) or P-ERK antibody (1:500 dillution; Cell Signaling) overnight at 4°C. A second antibody of sheep antimouse or donkey antirabbit conjugated with horseradish perioxidse (GE Healthcare, UK) was used at 1:10000 dilution. Protein expression was detected by ECL (Amersham).

### Chromatin immunoprecipitation (ChIP) assays

ChIP assays were carried out as described previously.(23) Briefly, cells were cross-linked with 1% *p*-formaldehyde for 10 minutes at room temperature. Chromatin then was isolated and sheared by sonication to produce an average fragment size of 300 to 500 bp. Chromatin (10 µg DNA/assay) was precleared by incubation with protein A/G agarose beads (Santa-Cruz Biotechnology) for 1 hour, incubated with primary antibodies to Runx2 or P-ERK, and precipitated with A/G agarose beads. Input DNA and DNA from ChIP samples were measured by PCR (35 cycles) or by quantitive real-time PCR using Taqman probes flanking OSE2a, OSE2b, or control regions in the mOG2 gene, as described previously.(23) All PCR primer sequences used in this study to measure interactions with *Bglap2* were described in Roca et al.,(23) whereas primers for *Ibsp* were described in Roca et al.(24) ChIP assays conducted using transfected wild-type and mutant 0.6mOG2-Luc plasmid used the following PCR primers: OSE2a 5' primer: 5'-CCTTGCCCAGGCAGCTGCAATCA-3'; OSE2a 3' primer: 5'-TCTTCCAGCGGATAGAATGGCGC-3'; OSE2b 5' primer: 5'-CTAGCAAAATAGGCTGTCCCCAG-3'; and OSE2b 3' primer: 5'-GCCTCCATAAGATCCGGTTGGTA-3'. ChIP-Re-ChIP analysis was conducted as described by Young et al.(25) Briefly, chromatin immunocomplexes initially isolated with anti-Runx2 antibody were disrupted with 10 mM DTT at 37°C for 30 minutes and then diluted with IP buffer. The diluted chromatin complexes were subjected to the second ChIP analysis with antibody against P-ERK.

### Immunofluorescence localization of Runx2 and P-ERK

MC3T3-E1c4 cells were grown on glass cover slips, fixed with 4% formaldehyde, and incubated overnight at 4°C with primary antibodies to Runx2 and P-ERK (1:100 dilution). Alexa Fluor 488 conjugated donkey antimouse and Alexa Fluor 555 conjugated donkey antirabbit (Invitrogen) antibodies were used to visualize the localization of Runx2 and P-ERK. Fluorescence detection was accomplished using an Olympus FluoView 500 laser scanning confocal microscope system with a 100× oil-immersion objective at room temperature (Microscopy and Image Analysis Laboratory, University of Michigan School of Medicine).

### Transient transfections and luciferase reporter assays

Luciferase reporter plasmids containing 610 bp of mOG2 promoter (0.6mOG2-Luc) with or without mutations in OSE2a and/or OSE2b sequences were described previously.(26) Transient transfection of MC3T3-E1c4 cells was accomplished using lipofectamine reagent (Invitrogen), and luciferse activity was measured using a Monolight A200 luminometer. *Runx2* expression in C3H10T1/2 cells was accomplished using a previously described adenovirus expression vector.(27)

### Statistical analysis

The results are presented as mean ± SE, with *n* = 3 per group for all comparisons. Statistical analysis was determined using a one-way ANOVA followed by Tukey's multiple-comparison test.

## Results

### MAP kinase signaling increases during ECM-induced osteoblast differentiation

**Fig. 1:**
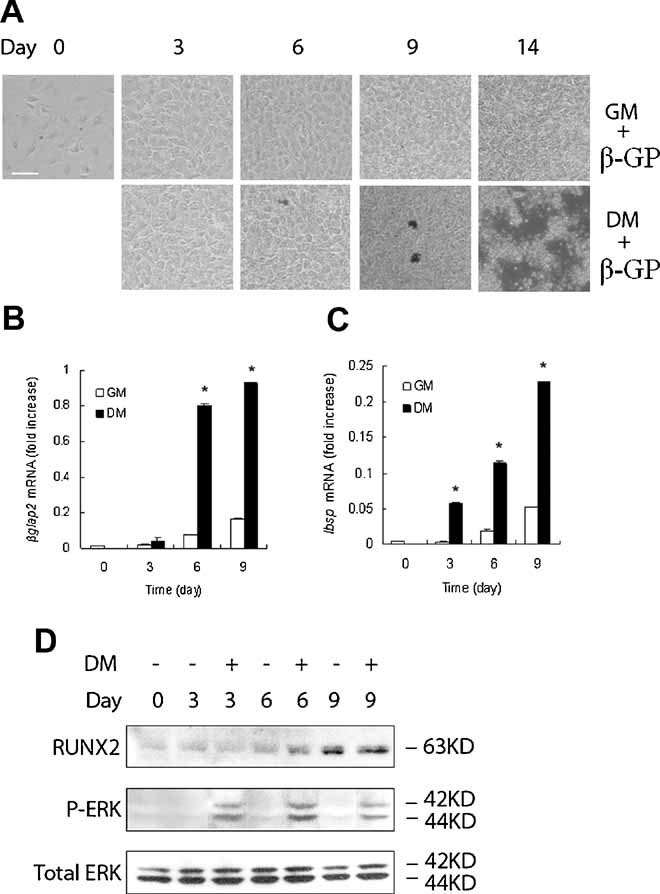
Time course of differentiation and MAP kinase activation in MC3T3-E1 clone 4 preosteoblast cells. (*A–D*) Differentiation. Cells were cultured in growth or differentiation medium (+DM) for the times indicated and either stained for mineral (*A*) or analyzed for changes in expression of *Bglap2* (*B*) or *Ibsp* genes (*C*). To assess mineralization, DM was supplemented with β-glycerol phosphate. Cells were stained by the von Kossa method and photographed using phase-contract microscopy (bar, 100 µm). For gene expression analysis, total RNA was isolated, and relative levels of *Bglap2* and *Ibsp* mRNA were measured by Q-RT-PCR. (*D*) Runx2 and phospho-ERK expression during osteoblast differentiation. The membrane/nuclear fractions of cell lysates were isolated at the times indicated and extracted into RIPA buffer for Western blot analysis of RUNX2, phospho-ERK, and total ERK. Significantly different from corresponding time-matched GM control, *p* < .01(*).

Previous work established a requirement for ERK–MAP kinase signaling in osteoblast differentiation in vitro and in vivo.(4,13,15) To examine the temporal relationship between MAP kinase activation and differentiation, MC3T3-E1c4 cells were cultivated in growth (GM) or differentiation medium (DM), and the time course of differentiation marker induction (extracellular matrix mineralization, *Bglap* and *Ibsp* gene expression) was compared with ERK phosphorylation (Fig. 1). DM, which contains ascorbic acid, stimulates secretion of a collagenous extracellular matrix that induces osteoblast differentiation by interacting with α2β1 integrins to stimulate ERK-MAPK signaling.(4,16,28) As expected, all three differentiation markers were induced after 3 to 6 days in DM and continued to increase at later time points (panels *A–C*). Similarly, P-ERK was detected in the crude nuclear/membrane fraction of cells after 3 days in DM and subsequent time points (panel *D*), whereas total ERK levels remained relatively constant. In contrast, cells maintained in GM did not mineralize, expressed only trace amounts of *Bglap* or *Ibsp* mRNA, and had very low levels of P-ERK. RUNX2 protein levels also were measured on Western blots (Fig. 1*D*). Although growth in DM dramatically increased expression of RUNX2 target genes, levels of RUNX2 protein were similar in GM- and DM-treated samples at each time point, as would be expected if ECM–MAP kinase signals were increasing RUNX2 transcriptional activity rather than amount.

### Differentiation-dependent accumulation of phosphorylated ERK in the nucleus and colocalization with RUNX2

**Fig. 2:**
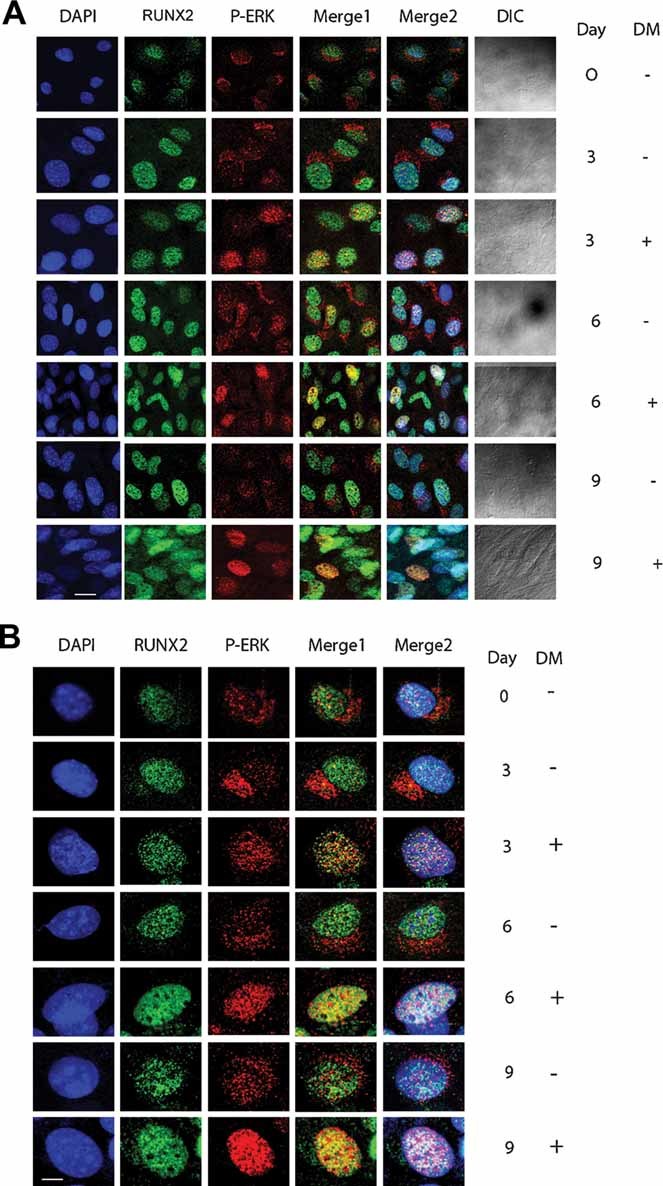
Colocalization of P-ERK and RUNX2 in osteoblast cells. MC3T3-E1 clone 4 cells were cultivated in GM or DM for the times indicated. After fixation, cells were incubated with DAPI, anti-RUNX2, and anti-P-ERK antibodies, followed by antimouse IgG conjugated with Fluo-Alex 488 and antirabbit IgG conjugated with Fluo-Alex 555. Colocalization was assessed by confocal microscopy. Merged pseudocolor images are shown in the indicated panels. Merge1 shows RUNX2 and P-ERK colocalization; merge2 combines merge1 with DAPI; DIC, differential interference contrast. (*A*) Lower-magnification view showing several cells/field. Scale bar, 20 µm. (*B*) Higher magnification showing subcellular localization in a single cell. Scale bar, 5 µm.

Immunofluorescence laser confocal microscopy was used to examine the intracellular location of P-ERK and RUNX2 during osteoblast differentiation (Fig. 2). Cells were grown in GM or DM and stained with specific anti-RUNX2 and anti-P-ERK antibodies or with DAPI to visualize nuclei. Low-power fields of cell cultures are shown in Fig. 2*A*, whereas higher magnifications of single cells are shown in Fig. 2*B*. Consistent with the Western blot results in Fig. 1, RUNX2 immunofluorescence increased gradually with time regardless of whether cells were cultured in GM or DM, but in all cases staining was exclusively nuclear. At each time point, all nuclei stained positively for RUNX2. Fluorescence intensity increased gradually from 4.5 ± 0.2 × 10^3^ pixels/nucleus at the beginning of the experiment to 10.6 ± 0.5 × 10^3^ pixels/nucleus at day 9 (*n* = 20 nuclei/time point). In contrast, the weak P-ERK immunofluorence seen in GM cultures was mainly in the cytosol, although some weak nuclear or perinuclear staining and colocalization with RUNX2 was seen occasionally. Growth in DM dramatically changed the distribution of P-ERK from the cytoplasm to the nucleus at all time points examined. Much of this nuclear P-ERK colocalized with RUNX2. Although some heterogeneity in P-ERK and, to a lesser extent, RUNX2 fluorescence was observed, generally, cells that exhibited strong nuclear P-ERK staining also showed strong colocalization of P-ERK with RUNX2. Another feature of P-ERK and RUNX2 nuclear distribution apparent in higher-power views is the punctate distribution of both antibodies. This has been proposed to reflect the localization of proteins to specific subnuclear domains.(29)

### P-ERK binds to specific regions of mouse osteocalcin gene 2 (Bglap2) chromatin

To more precisely define the nuclear location of P-ERK in differentiating osteoblasts, we used ChIP analysis to test the hypothesis that this kinase associates with specific regions of osteoblast-related genes. This model is based on work in other differentiating systems such as muscle showing that nuclear kinases can bind to chromatin near tissue-specific transcription factors such as *myoD*.(18)

**Fig. 3:**
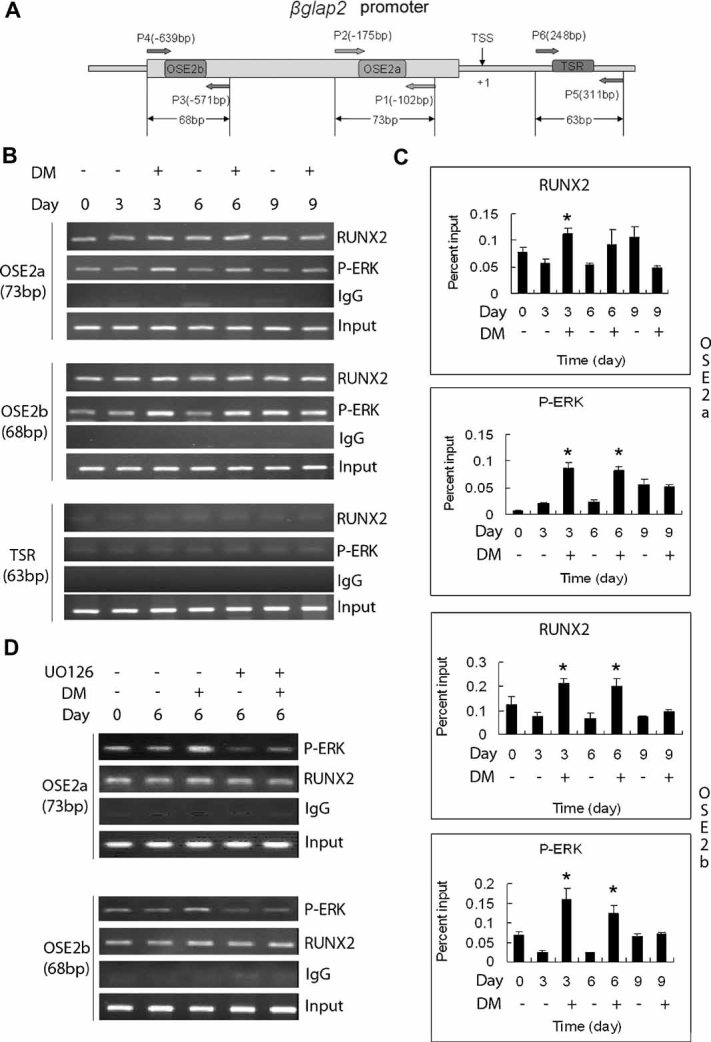
Association of RUNX2 and P-ERK with OSE2 elements in mOG2 chromatin during osteoblast differentiation. (*A*) Schematic of the proximal mOG2 promoter showing the location of PCR primer pairs for ChIP assays. Primer pairs P1/P2 and P3/P4 flank OSE2a and OSE2b regions, respectively, whereas pair P5/P6 amplifies DNA in the transcribed region (TSR) 413 bp downstream from primer P1. (*B*) ChIP analysis. MC3T3-E1 cells were grown under the conditions described in Fig. 1, and chromatin was isolated at the times indicated. Monoclonal anti-RUNX2, anti-P-ERK antibodies, or control IgG were used to immunoprecipitate chromatin, and ChIP DNA was analyzed by PCR using the primers indicated. (*C*) Quantitation of ChIP DNA by Q-PCR. Values are expressed as percent of input DNA. Note the preferential increase in P-ERK associated with OSE2a and OSE2b regions in cells grown in DM and lesser variation in bound Runx2. Significantly different from corresponding time-matched GM control, *p* < .01(*). (*D*) MAP kinase inhibition blocks P-Erk, but not Runx2 binding to OSE2-containing chromatin. Cells were grown in GM or DM for 6 days followed by treatment for an additional 12 hours in the presence or absence of the MAPK inhibitor, U0126. Chromatin then was isolated for ChIP analysis as in panel *B*.

Our intial studies analyzed interactions between P-ERK and *Bglap2* chromatin. The proximal promoter of this gene, shown schematically in Fig. 3*A*, contains two RUNX2 binding sites designated OSE2a and OSE2b at –130 and –605 bp upstream from the transcription start site, respectively. Both sites are necessary for the osteoblast-specific expression of this gene in vivo.(26) For ChIP analysis, primers were designed to individually detect immunoprecipitated chromatin containing OSE2a and OSEb regions as well as chromatin from a transcribed region (TSR) 400 bp downstream from OSE2a, which serves as a negative control. Previous work showed that OSE2a and OSE2b are sufficiently far apart (469 bp) to not be precipitated in the same chromatin fragment during ChIP analysis and can, therefore, be analyzed separately.(23) MC3T3-E1c4 cells were cultured in GM or DM, and ChIP analysis was performed using antibodies specific to RUNX2, P-ERK, or isotype-specific IgG (see Fig. 3*B, C*). Panel *B* shows representative PCR reactions of samples, whereas panel *C* quantifies relative amounts of ChIP DNA using real-time PCR. RUNX2 bound OSE2a and OSE2b chromatin to a similar degree, and the amount of binding was modestly increased by differentiation (maximum variation between GM and DM at 3 and 6 days was two- to threefold). Interestingly, P-ERK also bound to OSE2a and OSE2b, and this chromatin binding was strongly upregulated (five- to sixfold) by growth in DM for 3 or 6 days. However, we did not detect DM-dependent increases in P-ERK or RUNX2 binding at the 9-day time point. This is to be contrasted with immunofluorescence data showing nuclear accumulation of P-ERK and increased RUNX2 staining intensity at this time. The specificity of these interactions was confirmed in several ways (panel *B*): (1) Little or no PCR signal was obtained when ChIP DNA samples were analyzed using PCR primers specific to the downstream transcribed region of *Bglap2* (TSR). (2) No ChIP PCR product was obtained when chromatin was precipitated with control IgG. In this latter case, we used real-time PCR to detect any residual OSE2a and OSE2b sequences in IgG immunoprecipitates and found levels to be consistently below 0.0004 percent of input (not depicted). (3) P-ERK ChIP DNA samples did not contain detectable promoter sequences from the *Gapdh* gene, which is constitutively active in osteoblasts, indicating that P-ERK preferentially binds to *Bglap2* chromatin and does not generally associate with the promoter regions of transcribed genes (not depicted).

As shown in Fig. 3*D*, binding of P-ERK to *Bglap2* chromatin requires MAP kinase signaling. In this experiment, cells were grown for 6 days in GM or DM and then treated for 12 hours with or without the ERK–MAP kinase inhibitor, U0126, which is known to block *Bglap2* and *Ibsp* expression in MC3T3E1c4 cells.(4) The DM-treated group showed increased binding of P-ERK to OSE2a and OSE2b regions, as expected, and this response was completely blocked by U0126. In contrast, RUNX2 binding was not affected by inhibitor treatment. Results of this experiment were confirmed by real-time PCR of ChIP DNA samples using primers to the OSE2a region. Percent input values were as follows: 0-day control: 0.012 ± 0.006; 6-day control: 0.011 ± 0.002; 6-day DM: 0.036 ± 0.002; 6-day control + U0126: 0.0094 ± 0.001; 6-day DM + U0126: 0.010 ± 0.0055.

### Recruitment of P-ERK to Bglap2 chromatin requires intact OSE2 and RUNX2

**Fig. 4:**
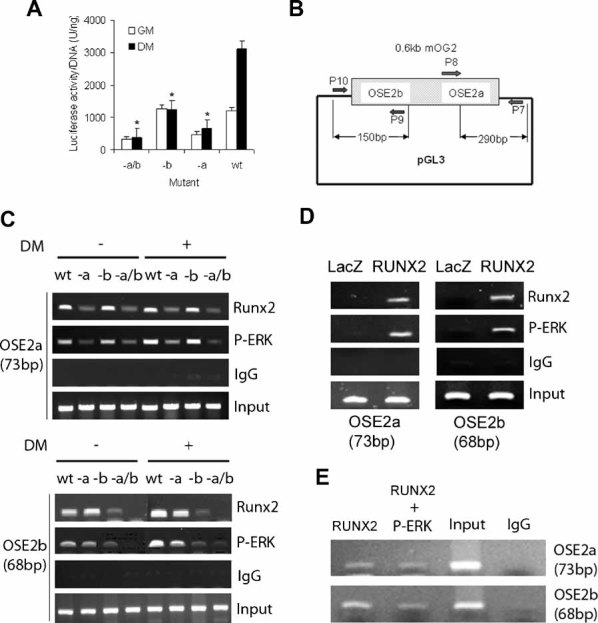
Association of P-ERK with mOG2 chromatin requires intact OSE2 regions and RUNX2. (*A*) Effect of *OSE2* mutations on mOG2 promoter activity. To confirm that OSE2a and OSE2b are required for activation of Ocn, MC3T3-E1c4 cells were transfected with wild-type 0.6mOG2-luc (wt) or 0.6mOG2-luc harboring point mutations in *OSE2a* (–a), *OSE2b* (–b), or both sites (–a/b) and assayed for luciferase activity after growth in GM or DM for 6 days. The *OSE2* mutant is significantly different from wild- type (DM samples only), *p* < .01(*). (*B*) Schematic showing location of PCR primers used for detecting interactions with *OSE2a* or *OSE2b* in WT or mutant 0.6mOG2-luc. (*C*) Mutation of *OSE2* sites block binding of RUNX2 and P-ERK to mOG2 chromatin. Cells were transfected with WT or mutant 0.6mOG2-luc and cultured in GM or DM as in panel *A*. Isolated chromatin fragments were precipitated with anti-RUNX2 and anti-P-ERK antibodies or control IgG and ChIP DNA analyzed by PCR using the primers indicated. (*D*) Runx2 is indispensable for binding of P-ERK to mOG2 chromatin. C3H10T1/2 mesenchymal cells were transduced with control adenovirus (AdLacZ) or an adenovirus expressing Runx2 (AdRunx2) and grown for 4 days before chromatin isolation and ChIP analysis. Note the lack of a positive ChIP signal for P-ERK in the absence of RUNX2. (*E*) ChIP-re-ChIP detection of RUNX2 and P-ERK on *Bglap2* chromatin. MC3T3-E1c4 cells were grown for 6 days as in panel *A*. ChIP was performed using anti-RUNX2 antibody. A portion of the immunoprecipitated chromatin complex was eluted from agarose beads and subjected to a second ChIP with either anti-P-ERK antibody or IgG. ChIP DNA then was analyzed using PCR primers specific to OSE2a and OSE2b (see Fig. 1*A*).

The above-described results show that P-ERK selectively binds to *Bglap2* chromatin near the RUNX2-specific enhancers OSE2a and OSE2b. In view of our previous work showing that ERK–MAP kinase stimulates osteoblast-specific gene expression via a RUNX2-dependent pathway,(4,15) we designed a set of experiments to resolve whether the binding of P-ERK to *Bglap2* is, in fact, OSE2- and RUNX2-dependent (Fig. 4).

The first study resolved whether intact OSE2 sequences are required for P-ERK binding. Cells were transfected with wild-type 0.6mOG2-Luc (contains 0.6 kb of the *Bglap2* promoter driving firefly luciferase) or the same vector containing mutations in OSE2a, OSE2b, or both sites. In agreement with previous work,(26,30) activity of this reporter is strongly induced by growth in DM for 5 days and highly dependent on intact OSE2a and OSE2b sequences (panel *A*). ChIP assays next were used to determine if OSE2a and OSE2b are necessary for binding of P-ERK to *Bglap2*. Although ChIP is used most often to examine chromatin interactions in the context of chromosomal DNA, it also can be used to examine interactions with transfected plasmid DNAs. This is possible because transfected DNA is assembled into nucleosomes resembling the normal chromatin structure.(31) For this experiment, specific PCR primers were designed to detect interactions with the 0.6mOG2-Luc plasmid without interference from the endogenous *Bglap2* gene (see Fig. 4*B*). To detect interactions with OSE2a, a 5' primer was designed complementary to sequences upstream of the OSE2a region, and a 3' primer was selected in the luciferase coding region. To detect OSE2b binding, the 5' primer was in the pGL3 plasmid sequence, and the 3' primer was slightly downstream of OSE2b. The results of ChIP assays are shown in panel *C*. Binding of both RUNX2 and P-ERK to both OSE2a and OSE2b regions of the wild-type 0.6mOG2-Luc plasmid was easily detected. In contrast, mutation of OSE2a reduced RUNX2 and P-ERK binding when the OSE2a-specific primers were used, whereas mutation of OSE2b was without effect. Similarly, mutation of OSE2b reduced binding of both proteins, as measured using the OSE2b primers, whereas the OSE2a mutation did not affect binding. Finally, mutation of both sites reduced ChIP signals detected by either primer pair. From these studies we conclude that binding of P-ERK to *Bglap2* chromatin requires intact RUNX2 binding sites.

To examine the requirement for RUNX2 in this response, ChIP analyis was conducted using C3H10T1/2 mesenchymal cells with or without transduction with a RUNX2-expressing adenovirus (ses Fig. 4*D*). These cells normally express very low levels of RUNX2,(27) and as can be seen, no RUNX2 or P-ERK was detected in complex with OSE2a or OSE2b when cells were transduced with a control AdLacZ virus. In contrast, AdRunx2 transduction led to positive ChIP reactions for both RUNX2 and P-ERK.

Final confirmation that RUNX2 and P-ERK reside on the same chromatin fragment came from ChIP/re-ChIP analysis (see Fig. 4*E*). For this experiment, MC3T3E1c4 cells were grown in DM for 6 days. ChIP was carried out initially with RUNX2 antibody. The immunoprecipitate then was spit into thirds. The first portion was used for PCR analysis of precipitated DNA (RUNX2), the second was resuspended and precipitated with anti-P-ERK (RUNX2+P-ERK), and the third was precipitated with IgG. As can be seen, the anti-RUNX2 immunoprecipitate was specifically reprecipitated with the P-ERK antibody.

### Binding of P-ERK and RUNX2 to the bone sialoprotein gene (Ibsp)

**Fig. 5:**
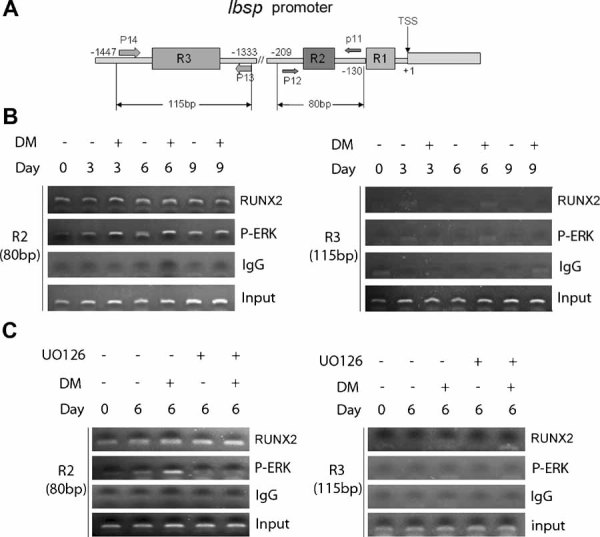
Association of RUNX2 and P-ERK with *Ibsp* chromatin during osteoblast differentiation. (*A*) Schematic of the *Ibsp* proximal promoter regions showing positions of PCR primers for ChIP analysis. The P7/P8 primer pair detects interactions with a proximal promoter region containing 2 RUNX2 binding sites, whereas P9/P10 detects interactions with a distal region devoid of in vivo RUNX2 interactions. (*B*) RUNX2 and P-ERK ChIP assays. Cells were grown as in Fig. 2. Note differentiation-dependent increases in P-Erk binding to the proximal promoter regions. (*C*) MAP kinase dependence of P-ERK binding.

*Ibsp* is a second major osteoblast-related gene that is upregulated during differentiation and bone formation (32) (see Fig. 1). Previous analysis of the murine gene promoter identified two RUNX2-specific enhancers (R1, R2) in the first 200 bp upstream from the transcription start site that were both shown to be necessary for osteoblast-specific expression.(24) A third nonfunctional RUNX2 consensus binding site also was identified at –1300 bp (see schematic in Fig. 5*A*). This site did not bind RUNX2 in ChIP assays and was devoid of enhancer activity. To evaluate the binding of P-ERK to *Ibsp*, MC3T3E1c4 cells were grown in GM or DM for increasing times, and ChIP analysis was carried out using specific primers flanking the R2 and R3 regions (see Fig. 5). Note that because R1 and R2 are only separated by approximately 100 bp, the p7/p8 primer pair cannot discriminate between binding to R1, R2, or both sites.(24) Consistent with results obtained with *Bglap2*, both RUNX2 and P-ERK bound to the R1/R2 region, whereas neither factor interacted with R3. Also, growth in DM clearly increased P-ERK binding after 3 or 6 days without having any noticeable effect on RUNX2 (panel *B*). Lastly, the differentiation-dependent increase in P-ERK binding seen on day 6 was preferentially blocked with MAP kinase inhibition without affecting RUNX2 (panel *C*). In summary, the specificity and MAP kinase dependence of P-ERK interactions with *Ibsp* are quite similar to those observed for *Bglap2*, making it highly likely that we are observing a general phenomenon related to the interaction of P-ERK with RUNX2 target genes.

## Discussion

In this study we examined the subcellular distribution of P-ERK during osteoblast differentiation and relate these changes to the known ability of ERK-MAPK signals to induce bone-specific gene expression. During differentiation, P-ERK accumulated in the nucleus, where it specifically bound to the chromatin of *Pglap2* and *Ibsp* genes near the RUNX2 transcription factor. P-ERK-chromatin binding required intact RUNX2-specific enhancer sequences in target genes, was detected only in RUNX2-expressing cells, and could be blocked by pharmacologic inhibition of ERK-MAPK signaling. As is clearly shown in this work, P-ERK does not have a random distribution in osteoblast nuclei. Instead, it directly interacts with target genes to facilitate their activation during differentiation.

Although previous studies established an important role for ERK-MAPK signaling in osteoblast differentiation, the mechanism of action of this pathway has not been explored extensively beyond showing that MAPK can increase transcriptional activity and phosphorylation of RUNX2.(4,13,16,17) To gain further insight into the role of MAPK signaling in osteoblast differentiation, we examined the detailed subcellular distribution of P-ERK. Undifferentiated MC3T3-E1c4 cells were found to contain low levels of P-ERK. This P-ERK was barely detectable on Western blots but was clearly visible by immunofluorescence confocal microscopy, where it was seen mainly in the cytosol. Interestingly, some of this cytosolic P-ERK was clustered in perinuclear regions, especially at early time points. Although we have not attempted to further localize this material, in other systems, part of the cellular P-ERK pool is retained in the cytoplasm in complex with the Golgi apparatus, where it phosphorylates cytoplasmic targets.(33) This cytoplasmic P-ERK pool may be required to maintain certain cellular functions in undifferentiated cells. When MC3T3-E1c4 cells were grown under differentiating conditions, we observed increases in both the amount of P-ERK relative to the total ERK pool, as well as a clear redistribution of P-ERK to the nucleus. In contrast, immunolocalization of the RUNX2 transcription factor revealed a similar punctate nuclear distribution in control and differentiating cells at all time points examined. Since P-ERK accumulated in the nucleus during differentiation, clear colocalization with RUNX2 became apparent.

Our novel finding that at least a portion of the nuclear P-ERK can be detected in complex with the chromatin of osteoblast-related genes is of particular interest and may begin to explain how MAP kinase can regulate gene expression. Both RUNX2 and P-ERK selectively associated with known RUNX2 enhancer regions of *Bglap2* and *Ibsp* genes. Furthermore, P-ERK and, to a lesser extent, RUNX2 binding was upregulated in differentiating cells at both day 3 and day 6 time points.

Surprisingly, growth in DM did not increase P-ERK binding to either gene at the day 9 time point even though immunofluorescence data revealed clear increases in nuclear P-ERK under this condition. This result may be explained by increases in the heterogeneity of osteoblast cultures during differentiation. Over time, cells form multilayered structures, with proliferating cells remaining at the medium–cell layer interface and more differentiated cells being incorporated into the collagenous matrix, where they initiate mineralization within collagen fibrils.(34,35) An example of this heterogeneity is shown in Fig. 2*A*. Although growth in DM clearly increased nuclear P-ERK, this was apparent only in a fraction of the total cells in each field, particularly at the later time points. Possibly, cells not containing nuclear P-ERK are diluting the ChIP assay signal under this condition.

We used a variety of approaches to establish the specificity of P-ERK-chromatin interactions. Significantly, P-ERK did not have a random distribution on either *Bglap2* or *Ibsp* genes. Instead, it associated only with regions near functional binding sites for RUNX2. Thus P-ERK associated with OSE2a and OSE2b regions of the in *Bglap2* promoter but not with a 3' distal sequence within the transcribed region. Similarly, it bound near R1/R2 RUNX2 enhancers in the proximal *Ibsp* promoter but not to a cryptic nonfunctional RUNX2 consensus site at –1300 bp. Furthermore, mutation of RUNX2 binding sites (OSE2a, OSE2b) in the *Bglap2* promoter prevented both P-ERK and RUNX2 from associating with chromatin. Lastly, P-ERK was unable to associate with these chromatin regions in RUNX2-negative C3H10T1/2 cells, but binding could be restored with RUNX2 overexpression.

Our results with P-ERK and RUNX2 are reminiscent of previous observations related to muscle regeneration. This process is cooperatively induced by p38 MAPK and P13K/AKT pathways in response to acute injury.(18,36) In this case, the muscle-related transcription factors MyoD and MEF2 recruit p38α/β kinases to the promoters of muscle-related genes such as myogenin and muscle creatine kinase. Subsequent p38-dependent phosphorylation events are necessary for recruitment of the SWI/SNF chromatin-remodeling complex. Through a separate pathway, AKT promotes the association of MYOD with p300 and PCAF acetyltransferases. The resulting changes in acetylation convert the chromatin of myogenic genes into an open conformation that is permissive for transcription.

Subsequent reports described the association of nuclear kinases with chromatin in a number of other tissues. For example, after exposure of pancreatic β-cells to elevated glucose concentrations, P-ERK binds to promoter regions of several glucose-inducible genes, including the insulin gene, where it recruits accessory transcription factors, including NFAT, MafA, and PDX-1.(37) Also, activation of mouse mammary tumor virus (MMTV) transcription by progesterone involves formation of a complex containing the progesterone receptor, ERK and the ERK substrate mitogen and stress-activated kinase 1 (MSK1), on the MMTV promoter followed by increased histone H3 phosphorylation, formation of a chromatin remodeling complex, and increased transcription.(38) Lastly, in cortical neurons stimulated with nerve growth factors and cardiomyocytes, polyADP-ribose polymerase 1 (PARP-1) was shown to recruit P-ERK to the nucleus, leading to phosphorylation/activation of the ERK substrate Elk1, CBP/p300 phosphorylation, and increased histone acetylation.(39) Perhaps most interestingly, studies in yeast indicate that kinase occupancy of specific chromatin sites may be a common mechanism for controlling gene expression in eucaryotes.(19) In these studies, ChIP-ChIP analysis was used to show that, after activation, the yeast homologues of p38, ERK-MAPK, and PKA all become associated with the chromatin of multiple target genes to globally alter patterns of gene expression in response to osmotic stress, pheromones, or glucose exposure. Based on these studies, it was proposed that nuclear kinases generally may function by associating with multiple chromatin sites to globally alter gene expression.

The extent to which these concepts are applicable to bone is currently unknown. The predominant role of MAPK on osteoblast chromatin may be to phosphorylate RUNX2, or it may have additional, more global functions. As noted earlier, there is some precedent for the concept that tissue-specific transcription factors including myoD, PPARγ, and Sox9 are regulated by MAPK-dependent phosphorylation,(11,12,14) and as was shown by this laboratory, the ERK-MAPK pathway does, in fact, stimulate RUNX2 phosphorylation and transcriptional activity in osteoblasts.(13) We recently identified specific serine residues in the C-terminal proline/serine/threonine-rich domain of RUNX2 that are phosphorylated by MAPK and necessary for MAPK responsiveness (C Ge, G Xiao, Y Li, and RT Franceschi, submitted for publication). Furthermore, we were able to detect interactions between RUNX2 and P-ERK by coimmunoprecipitation. This latter results suggest that the association of P-ERK with RUNX2-binding regions of *Bglap2* and *Ibsp* genes may be explained by direct binding of this kinase to RUNX2.

However, in addition to being a potential P-ERK substrate, RUNX2 also may function to target nuclear kinases to specific chromatin regions, where they can have broader effects on chromatin structure and transcription. The concept that RUNX2 can serve as part of a nuclear scaffold to sequester specific factors within the nucleus was first proposed by the Stein/Lian laboratory largely based on studies showing that RUNX factors are components of the nuclear matrix and can target SMAD proteins to specific nuclear regions.(29) As noted earlier, chromatin-associated kinases can trigger multiple events, including recruitment and phosphorylation of other transcription factors and histones, activation of secondary nuclear kinases, and stimulation of histone acetylation and changes in chromatin structure. It is therefore conceivable that nuclear kinases have similarly complex roles during osteoblast differentiation. Increases in histone H3 and H4 acetylation have been correlated previously with differentiation in primary osteoblasts, although the possible involvement of nuclear kinases in this process has not been addressed.(40) In preliminarly studies, we detected increases in histone H3 and H4 acetylation during differentiation of MC3T3-E1c4 cells and found that these increases could be blocked with MAP kinase inhibition (not depicted), suggesting that one of the functions of nuclear P-ERK in osteoblasts may be to regulate acetylation.

Although only *Pglap2* and *Ibsp* interactions were examined in the present study, we think it likely that P-ERK and other nuclear kinases are associated with additional genes necessary for induction and maintenance of the osteoblast phenotype. The differentiation of osteoblasts from mesenchymal precursors requires both the induction of genes necessary for maintenance of the osteoblast phenotype and suppression of genes associated with other mesenchymal lineages. Such control requires a coordinated mechanism to simultaneously regulate transcriptional activity at multiple sites. By associating with osteoblast-related genes, nuclear kinases are ideally positioned to provide this type of global control. This is a plausible mechanism given that many of the major signals affecting osteoblast differentiation (i.e., hormones/growth factors, extracellular matrix, or mechanical loading) use MAP kinase–related pathways to convey information from the cell surface to the nucleus. However, the extent to which these concepts can be applied generally to bone will require analysis of kinase interactions with other osteoblast-related genes and associated transcription factors in the presence of different osteogenic signals. This analysis will be critical for mapping the transcriptional networks activated as bone responds to a range of environmental cues.
